# Function and Regulation of Human Terminal Uridylyltransferases

**DOI:** 10.3389/fgene.2018.00538

**Published:** 2018-11-12

**Authors:** Yuka Yashiro, Kozo Tomita

**Affiliations:** Department of Computational Biology and Medical Sciences, Graduate School of Frontier Sciences, The University of Tokyo, Kashiwa, Japan

**Keywords:** terminal uridylyltransferase, TUTase, TUT1, TUT4/7, U6 snRNA, *let-7*, biogenesis, splicing

## Abstract

RNA uridylylation plays a pivotal role in the biogenesis and metabolism of functional RNAs, and regulates cellular gene expression. RNA uridylylation is catalyzed by a subset of proteins from the non-canonical terminal nucleotidyltransferase family. In human, three proteins (TUT1, TUT4, and TUT7) have been shown to exhibit template-independent uridylylation activity at 3′-end of specific RNAs. TUT1 catalyzes oligo-uridylylation of U6 small nuclear (sn) RNA, which catalyzes mRNA splicing. Oligo-uridylylation of U6 snRNA is required for U6 snRNA maturation, U4/U6-di-snRNP formation, and U6 snRNA recycling during mRNA splicing. TUT4 and TUT7 catalyze mono- or oligo-uridylylation of precursor *let-7* (*pre–let-7*). *Let-7* RNA is broadly expressed in somatic cells and regulates cellular proliferation and differentiation. Mono-uridylylation of *pre–let-7* by TUT4/7 promotes subsequent Dicer processing to up-regulate *let-7* biogenesis. Oligo-uridylylation of *pre–let-7* by TUT4/7 is dependent on an RNA-binding protein, Lin28. Oligo-uridylylated *pre–let-7* is less responsive to processing by Dicer and degraded by an exonuclease DIS3L2. As a result, *let-7* expression is repressed. Uridylylation of *pre–let-7* depends on the context of the 3′-region of *pre–let-7* and cell type. In this review, we focus on the 3′ uridylylation of U6 snRNA and *pre-let-7*, and describe the current understanding of mechanism of activity and regulation of human TUT1 and TUT4/7, based on their crystal structures that have been recently solved.

## Introduction

Modification of the 3′-end of RNA by template-independent nucleotide addition is a post-transcriptional modification that plays important regulatory roles in gene expression. A well-known example of 3′-end modification is the addition of CCA to the 3′-end of tRNA at positions 74–76 by CTP:(ATP)-tRNA nucleotidyltransferase (CCA-adding enzyme) and related enzymes (Deutscher, 1990; Tomita and Weiner, 2001, 2002; Weiner, 2004). CCA-addition to the 3′-end of tRNA is required for amino acid attachment to the 3′-terminus of tRNA by aminoacyl-tRNA synthetases (Sprinzl and Cramer, 1979), and also for peptide bond formation on the ribosome (Green and Noller, 1997; Kim and Green, 1999; Nissen et al., 2000). Further, CCA-addition to the 3′-end of tRNA is involved in the quality control of dysfunctional tRNAs. Dysfunctional tRNA molecule with an unstable acceptor stem is modified by CCACCA addition, and the CCACCA tail serves as a degradation signal for cellular RNA decay machinery (Wilusz et al., 2011; Betat and Morl, 2015; Kuhn et al., 2015). Another well-known example of template-independent nucleotide addition to the 3′-end of RNA is polyadenylation of mRNA by a canonical PAP. Polyadenylation of mRNA regulates mRNA stability, mRNA export from the nucleus to cytoplasm, and translation initiation in eukaryotes (Beelman and Parker, 1995; Sachs et al., 1997; Wahle and Rüegsegger, 1999; Edmonds, 2002; Moore and Proudfoot, 2009). Polyadenylation of mRNA also regulates degradation of mRNA in eubacteria (Carpousis et al., 1999; Dreyfus and Régnier, 2002; Régnier and Hajnsdorf, 2009).

Detailed mechanism of polyadenylation by canonical PAPs (Bard et al., 2000; Martin et al., 2000; Balbo and Bohm, 2007), and that of CCA-addition by CCA-adding enzymes and related enzymes have been clarified in the last two decades (Li et al., 2002; Okabe et al., 2003; Xiong et al., 2003; Tomita et al., 2004, 2006; Xiong and Steitz, 2004, 2006; Toh et al., 2008, 2009, 2011; Pan et al., 2010; Tomita and Yamashita, 2014; Yamashita et al., 2014, 2015; Yamashita and Tomita, 2016). However, a new family of PAPs, non-canonical PAPs, have emerged, with the fission yeast cytoplasmic PAP, Cid1, first identified as a non-canonical PAP (Wang et al., 2000), which was later revealed to be a terminal uridylyltransferase (Rissland et al., 2007). Non-canonical PAPs are conserved and play important roles in gene expression in various eukaryotes, from yeast to human (Stevenson and Norbury, 2006; Norbury, 2010; Scott and Norbury, 2013; Lee et al., 2014; De Almeida et al., 2018). Phylogenetic distribution of non-canonical PAPs in eukaryotes has recently described (Chang et al., 2018). The family of proteins share the catalytic domain with canonical PAPs but contain different ribonucleotide base recognition motifs (Martin and Keller, 2007). As a result, some of the non-canonical PAPs bearing histidine insertion in the ribonucleotide base recognition motif use UTP as a substrate and function as TUTases (Kwak and Wickens, 2007; Rissland et al., 2007; Mullen and Marzluff, 2008; Wickens and Kwak, 2008).

Various classes of RNAs, including mRNA, miRNA and snRNA, are uridylylated by non-canonical terminal nucleotidyltransferase family of enzymes. In Trypanosome mitochondria, uridylylation is required for guide RNA maturation (Aphasizhev et al., 2016). Uridylylation is also important for regulation of small RNA expression. In *Drosphila melanogaster*, a TUTase named Tailor prevents biogenesis of mirtron (Bortolamiol-Becet et al., 2015; Reimao-Pinto et al., 2015; Rissland, 2015), while uridylation serves as a degradation marker for small RNAs in various organisms (De Almeida et al., 2018). In addition, uridylylation also facilitates mRNA decay. Uridylylation-mediated mRNA degradation contributes to cellular mRNA metabolism and also is involved in maternal mRNA clearance during maternal to zygotic transition (Scott and Norbury, 2013; Lee et al., 2014; Lim et al., 2014; Morgan et al., 2017; Chang et al., 2018). Thus, uridylylation of RNA 3′-ends plays a pivotal role in the biogenesis and metabolism of functional RNAs, facilitating regulation of gene expression. The detailed functions of uridylylation were recently reviewed (De Almeida et al., 2018; Menezes et al., 2018).

**FIGURE 1:**
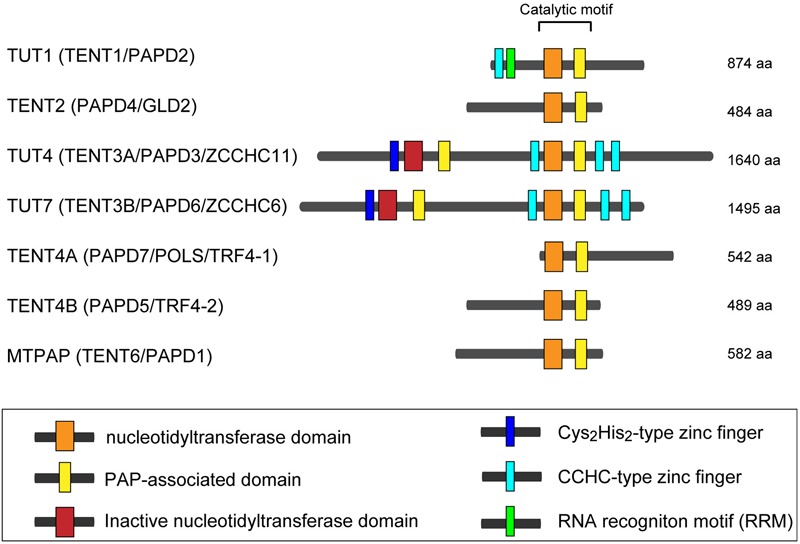
Human non-canonical terminal nucleotidyltransferases. Schematic representation of domain organization of seven human non-canonical terminal nucleotidyltransferases. The catalytic motif is composed of nucleotidyltransferase domain (orange box) and PAP-associated domain (yellow box). Inactive nucleotidyltransferase domains are designated by red boxes. C_2_H_2_-type zinc finger and CCHC zinc finger domains are designated as dark blue and light blue boxes, respectively. RNA recognition motif (RRM), is shown as a green box. The figure is modified from Heo et al. (2009) and Lee et al. (2014).

In human, seven non-canonical nucleotidyltransferases have been identified, with diverse cellular functions (Stevenson and Norbury, 2006; Martin and Keller, 2007; Wilusz and Wilusz, 2008). In this review, we use the updated HUGO-approved nomenclature to refer those enzymes, as HUGO-approved gene symbols for those non-canonical terminal nucleotidyltransferases have been recently changed (Figure 1). Among the seven human non-canonical terminal nucleotidyltransferases, four enzymes show adenylyltransferase activity. MTPAP is a mitochondorial PAP, which regulates stability of mitochondrial mRNAs (Tomecki et al., 2004; Nagaike et al., 2005). TENT2 adenylates selected mRNAs and miRNAs in cytoplasm (Kwak et al., 2004; Nagaike et al., 2005; Katoh et al., 2009; Glahder and Norrild, 2011; D’Ambrogio et al., 2012), while TENT4A and TENT4B add poly(A) to various classes of nuclear RNAs and involve in RNA degradation as a subunit of a TRAMP-like complex (Berndt et al., 2012; Ogami et al., 2013; Sudo et al., 2016). TENT4A and TENT4B have also recently been shown to be responsible for mRNA guanylylation (Lim et al., 2018).

The other three enzymes (TUT1, TUT4, and TUT7) are the TUTases that mediate template-independent uridylylation at the 3′-end of RNAs in the human cells. TUT1 is a nuclear TUTase and required for maturation process of the 3′-end of U6 snRNA (Scott and Norbury, 2013; Lee et al., 2014; Lim et al., 2014). On the other hand, TUT4 and TUT7 mainly localize in cytoplasm, and they are involved in various cellular processes, including regulation of miRNA biogenesis, surveillance for defective noncoding RNAs, replication dependent decay of poly(A)- histone mRNAs, and degradation of poly(A) + mRNAs (De Almeida et al., 2018; Menezes et al., 2018). In addition to those regulatory roles, TUT4 and TUT7 are also reported to uridylylate viral RNAs and LINE-1 mRNAs and act as immune system against genomic invasion (Le Pen et al., 2018; Warkocki et al., 2018; Yeo and Kim, 2018).

Recently, the crystal structures of human TUTases, TUT1 and TUT7, have been reported, and together with the biochemical studies of these enzymes, the molecular bases of uridylylation of 3′-end of specific RNAs have been proposed (Faehnle et al., 2017; Yamashita et al., 2017). In the current review, we describe the molecular mechanism and regulation of uridylylation of specific RNAs by human TUT1 and TUT7, based on their structures.

## TUT1: Oligouridylylation of U6 snRNA

### Biogenesis of U6 snRNA

Pre-mRNA splicing in eukaryotes is catalyzed by the spliceosome composed of five small ribonucleoprotein complexes (U1, U2, U4, U5, and U6 snRNPs) and a large number of proteins (Will and Luhrmann, 2011). U6 snRNP is composed of U6 snRNA, p110 (hPrp24), and heteroheptameric Lsm2–8 ring proteins. Proteins p110 and Lsm2–8 promote the annealing of U6 and U4 snRNAs for U4/U6 di-snRNP formation (Jandrositz and Guthrie, 1995; Raghunathan and Guthrie, 1998; Achsel et al., 1999). U5 snRNP joins the U4/U6 di-snRNP to form U4/U6∙U5 tri-snRNP. The U4/U6∙U5 tri-snRNP is recruited to the pre-spliceosome, composed of pre-mRNA, and U1 and U2 snRNPs. U6 snRNA forms an alternative helix with the U2 snRNA, following which two-step splicing reaction proceeds, accompanying the structural rearrangements of U6 snRNA in the spliceosome. In base-paired U6-U2 snRNAs, U6 snRNA participates in active-site formation and divalent cation coordination for the catalysis of splicing (Fica et al., 2013).

**FIGURE 2:**
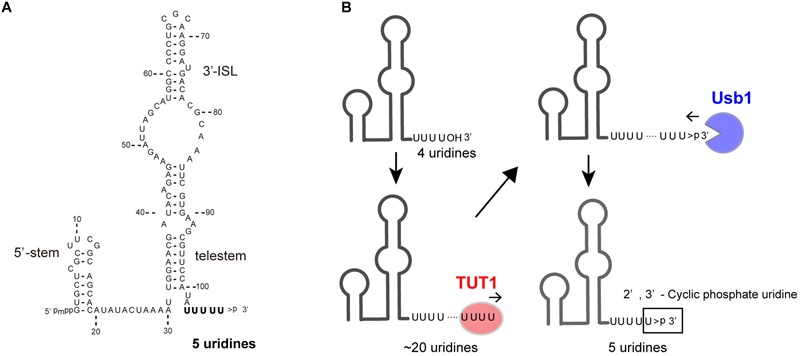
TUT1 in the maturation process of human U6 snRNA. **(A)** Secondary structure of human mature U6 snRNA transcript. Mature U6 snRNA harbors 5′-γ-methyl tri-phosphate (5′-pmpp) and 2′,3′-cyclic phosphate ( > p) at 5′- and 3′-ends, respectively. **(B)** Maturation of U6 snRNA. Primary U6 snRNA transcript harbors four genome-encoded 3′-uridines (UUUUOH). The 3′-end is oligo-uridylylated by TUT1, with the addition of up to 20 uridines. Finally, oligo-uridylylated U6 snRNA is trimmed by Usb1. Mature U6 snRNA harbors five 3′-uridines capped with a 2′,3′-cyclic phosphate (UUUUU > p).

U6 snRNA is transcribed by RNA polymerase III and undergoes multiple maturation processes (Wilusz and Wilusz, 2013). The U6 snRNA transcript has a 5′-stem, ISL, and telestem secondary structures (Rinke and Steitz, 1985; Karaduman et al., 2006; Figure 2A). The U6 snRNA primary transcript contains four genome-encoded 3′-end uridines (U4-OH) (Figure 2B). After transcription, the 3′-end is oligo-uridylylated by TUT1 (Trippe et al., 1998; Trippe et al., 2006). Then, the oligo-uridylylated tail of U6 snRNA is trimmed by a 3′–5′ exonuclease, Mpn1 (Usb1) (Mroczek et al., 2012; Shchepachev et al., 2012; Hilcenko et al., 2013). The 3′-end of the mature U6 snRNA has five uridines capped with a 2′,3′-cyclic phosphate (U4–U > p), which protects U6 snRNA from degradation.

The oligo-uridylylated tail of U6 snRNA is the binding site for the Lsm2–8 complexes (Achsel et al., 1999; Vidal et al., 1999); for the annealing of U6 and U4 snRNAs to form di-U4/U6 snRNP; and for the recycling of U6 snRNA after the splicing reaction (Bell et al., 2002). Thus, 3′-oligo-uridylylation of U6 snRNA by TUT1 contributes to efficient pre-mRNA splicing in cells. Human TUT1 was originally identified as a U6 snRNA–specific TUTase (Trippe et al., 2003; Trippe et al., 2006). Subsequently, it was also reported that TUT1 can function as a PAP acting with specific mRNAs under specific conditions (Mellman et al., 2008).

### Structure of Human TUT1

Recently, the crystal structures of human TUT1, and its complexes with UTP or ATP have been reported (Yamashita et al., 2017). These were the first structures of a TUTase from a higher eukaryote. Human TUT1 is a multi-domain protein composed of an N-terminal ZF, N-terminal RRM, a catalytic motif in the middle, and an uncharacterized C-terminal domain (Trippe et al., 2006). The catalytic motif is composed of nucleotidyltransferase domain and PAP-associated domain (Figure 1). Since crystals of full-length human TUT1 protein could not be obtained, truncated forms of TUT1 protein were crystallized and their structures were determined.

**FIGURE 3:**
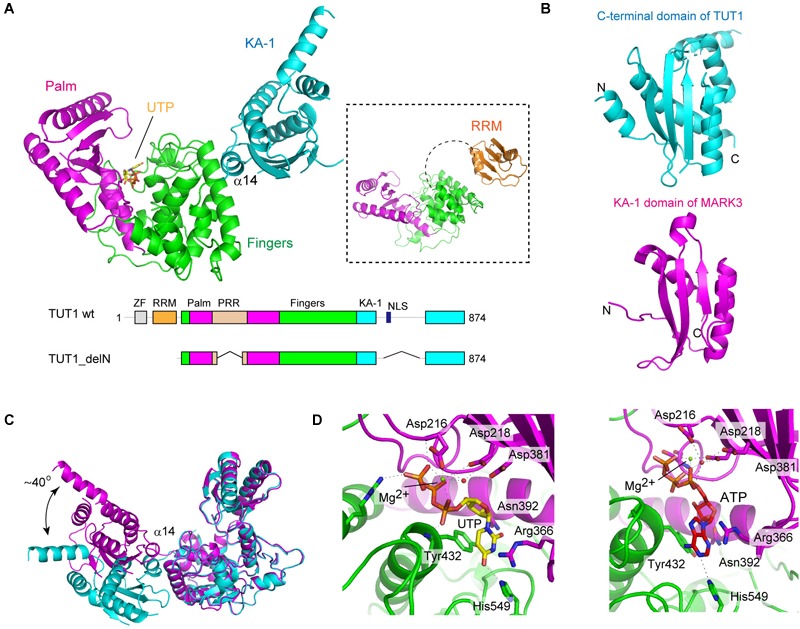
Structure of human TUT1. **(A)** The overall structure of human TUT1 lacking N-terminal ZF and RRM (TUT1_delN). Palm (magenta), fingers (green) and KA-1 (cyan). UTP (stick model in yellow) resides in the cleft between palm and finger domains. Inset, the overall structure of human TUT1 lacking C-terminal KA-1. The linker between RRM (orange) and palm domain is flexible. **(B)** KA-1 domain of TUT1 (upper, cyan) is homologous to KA-1 domain of MARK3 kinase (lower, pink). **(C)** Superposition of TUT1_delN structures of different forms of crystals. C-terminal KA-1 (cyan) domains of TUT1 are mobile and can rotate approximately 40 degrees relative to the catalytic domain, using α14 as the rotation axis. **(D)** Nucleotide recognition by human TUT1. UTP recognition (left) and ATP recognition (right). Nucleotides are depicted by stick models.

TUT1 (TUT1_delN), lacking N-terminal ZF and RRM, consists of three domains: the catalytic palm and finger domains, and an additional distinct domain linked to the C-terminus of the protein (Figure 3A). The C-terminal region of TUT1 is the previously unidentified RNA-binding domain, named KA-1 domain.

The overall structure of the catalytic core palm and finger domains of TUT1 shares topological homology with those of yeast Cid1 and vertebrate mitochondrial PAP (Bai et al., 2011; Lunde et al., 2012; Munoz-Tello et al., 2012; Yates et al., 2012; Lapkouski and Hallberg, 2015). The palm domain of human TUT1 consists of five-stranded β-sheets and two α-helices, and three catalytic carboxylates (Asp216, Asp218, and Asp381). The structure of TUT1 palm domain shares homology with those of DNA polymerase β family proteins (Aravind and Koonin, 1999). The finger domain has a helical structure with ten α-helices and three α-sheets, and is homologous to the central domain of PAPα (Bard et al., 2000; Martin et al., 2000). The incoming nucleotide is located in the cleft between the palm and fingers.

The C-terminal domain of TUT1 consists of four anti-parallel β-sheets and five α-helices (Figure 3B). This domain shares topological homology with the KA-1 domain of various proteins (Moravcevic et al., 2010). Structure of another crystal form of TUT1_delN suggests that the KA-1 domain can rotate by approximately 40 degrees with respect to the catalytic core domains, using α14 as the axis of rotation (Figure 3C). In the TUT1 structure lacking C-terminal KA-1 and N-terminal ZF domains, the N-terminal RRM adapts a typical RRM fold (Kenan et al., 1991), with four anti-parallel β-sheets stacked onto two α-helices. The RRM is connected to the catalytic domain by a flexible linker (Figure 3A). Thus, the N-terminal RRM and ZF are mobile in the RNA substrate-free form of TUT1.

### Nucleotide Recognition by TUT1

The structures of TUT1 in complex with either UTP or ATP have been reported (Figure 3D). Both UTP and ATP reside in the cleft between the palm and finger domains. In the structure of UTP-bound TUT1, the uracil base is sandwiched between Tyr432 and the side chain of Arg366. The O_2_ and O_4_ atoms of UTP form hydrogen bonds with Asn392 and His549, respectively. The N_3_ atom of UTP forms a hydrogen bond with a water molecule that also forms a hydrogen bond with Asp543. In the ATP-bound structure, only the N_1_ atom of the adenine base of ATP forms a hydrogen bond with His549. The mechanism of nucleotide recognition by TUT1 and the specificity of TUT1 are essentially the same as those of yeast Cid1 (Lunde et al., 2012; Munoz-Tello et al., 2012; Yates et al., 2012). Human TUT1 incorporates UMP more efficiently than AMP into U6 snRNA transcript ending with four uridines. The steady-state kinetics of nucleotide incorporation into U6 snRNA indicate that UTP is a much better substrate of TUT1 than ATP (around 700-fold) (Yamashita et al., 2017).

### Domain Requirement for U6 snRNA Recognition by TUT1

The structure of human TUT1-U6 snRNA complex is not yet available. However, recent biochemical studies using full-length and truncated human TUT1 variants suggest that U6 snRNA is recognized by multiple domains of TUT1 (Yamashita et al., 2017). Human TUT1 possesses additional domains compared with the yeast Cid1 structure. TUT1 is composed of N-terminal ZF, RRM, palm, finger, and KA-1 domains (Figures 1, 3A). The domain organization of TUT1 is also different from those of other human non-canonical terminal nucleotidyltransferase families, although the structures of catalytic domains are homologous (Stevenson and Norbury, 2006; Martin and Keller, 2007; Wilusz and Wilusz, 2008).

Steady-state kinetics revealed that human TUT1 variants lacking the N-terminal ZF domain (ΔZ), lacking both the ZF and RRM domains, (ΔZR), or lacking the KA-1 domain (ΔKA-1) exhibit reduced uridylylation of U6 snRNA transcript. The *K*_m_ values of U6 snRNA for ΔZ and ΔZR are ca. 5-folds higher than that for wild-type TUT1. The overall uridylylation efficiencies of ΔZ and ΔZR are less than 0.2% that of wild-type TUT1, and their reduced activities are associated with reduced catalytic efficiencies. Thus, the N-terminal ZF and RRM domains might assist in the proper positioning of the 3′-end of U6 snRNA within the catalytic site for catalysis. The *K*_m_ value of U6 snRNA for ΔKA-1 is about 10-folds higher than that of wild-type TUT1, and the overall uridylylation efficiency of ΔKA-1 is ca. 20% that of wild-type TUT1. Hence, the C-terminal KA-1 domain increases TUT1 affinity for U6 snRNA at the UMP-incorporation stage.

**FIGURE 4:**
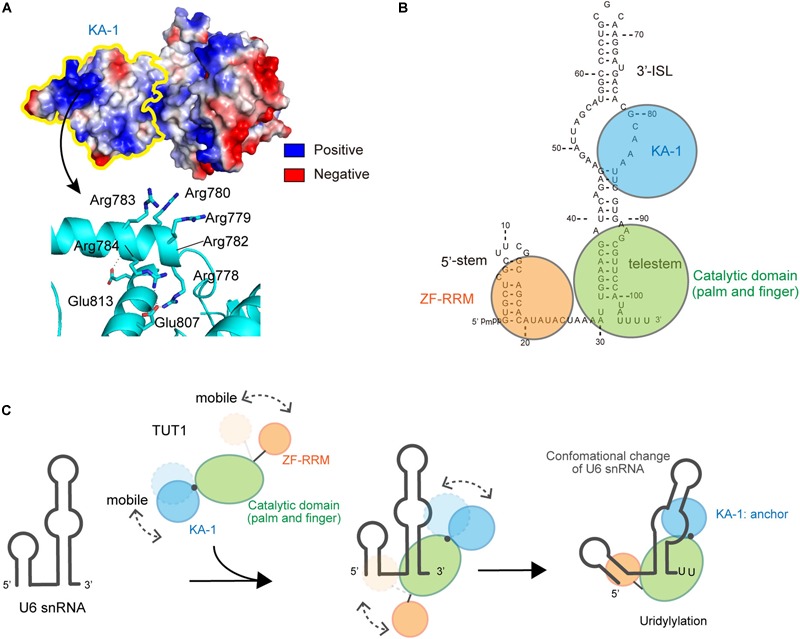
Interactions between U6 snRNA and TUT1. **(A)** Electrostatic potential of KA-1 domain of human TUT1. Positively and negatively charged areas are colored blue and red, respectively. KA-1 domain is outlined by yellow line (upper), and harbors clusters of positively charged amino acids (below). **(B)** Multi-domain utilization by TUT1 for U6 snRNA oligo-uridylylation. Schematic representation of interactions between U6 snRNA and TUT1 analyzed by Tb(III) hydrolysis mapping. N-terminal ZF and RRM (orange), catalytic palm and fingers (green), and C-terminal KA-1 (cyan). **(C)**. Mechanism of oligo-uridylylation of U6 snRNA by TUT1. KA-1 and ZF-RRM are mobile. Binding of TUT1 to U6 snRNA induces conformational change of U6 snRNA, and KA-1 of TUT1 acts as an anchor during oligo-uridylylation. N-terminal ZF and RRM (orange), catalytic palm and fingers (green), and C-terminal KA-1 (cyan).

The KA-1 domain of TUT1 is conserved among vertebrates, with positively charged clusters on the KA-1 surface (Figure 4A). The KA-1 domain itself is able to bind RNA, and substitutions of positively charged amino acids in the KA-1 domain to alanine reduce or abolish the RNA-binding activity. Thus, the previously unidentified C-terminal domain, KA-1, is an RNA-binding domain involved in U6 snRNA recognition, together with the N-terminal ZF and RRM domains. The N-terminal RRM is mobile relative to the catalytic core domains, and the C-terminal KA-1 rotates relative to the catalytic core domains (Figures 3A,C). Thus, at the UMP-incorporation stage, the domain movements would be coupled with the recognition of U6 snRNA.

### Interaction Between TUT1 and U6 snRNA, and Oligo-Uridylylation

TUT1 tightly interacts with U6 snRNA *in vivo*. The interactions between U6 snRNA and TUT1, and TUT1 truncated variants were recently analyzed by using Tb(III) hydrolysis mapping (Walter et al., 2000), and the protection patterns for U6 snRNA in the presence and absence of TUT1 protein and its variants were assessed. These studies demonstrated the TUT1 domain requirements for U6 snRNA recognition, as well as structural changes of U6 snRNA upon TUT1 binding.

U6 snRNA is recognized by multiple domains of TUT1 (Figure 4B; Yamashita et al., 2017). The N-terminal ZF and RRM domains of TUT1 interact with the single-stranded 5′-end of U6 snRNA, and the KA-1 domain interacts with the bulging loops. The core catalytic domain binds tightly to the double-stranded telestem region, and the 3′-region of U6 snRNA remains single-stranded. Almost the entire U6 snRNA sequence is recognized by the mobile N-terminal RNA-binding domain and the C-terminal KA-1 domain, cooperatively with the catalytic core domain. The recognition of U6 snRNA by TUT1 is coupled with domain movements and structural changes of U6 snRNA. In particular, interaction with TUT1 induces conformational changes in the 3′-ISL and the bulging loop of U6 snRNA.

The presence of N-terminal and C-terminal RNA-binding domains prevents U6 snRNA from dislodging from the enzyme surface during uridylylation reaction (Figure 4C). The C-terminal KA-1 of TUT1 might function as an anchor of the U6 snRNA molecule during oligo-uridylylation. TUT1 lacking C-terminal KA-1 or protein variants with substitutions of the positively charged residues in KA-1 (Figure 4A) add a relatively small number of UMPs (–2 nts) compared with wild-type TUT1 (–5 nts) (Yamashita et al., 2017). Absence of the KA-1 domain or loss of KA-1 RNA-binding activity would allow U6 snRNA to translocate easily on the enzyme surface. Following incorporation of several UMP molecules at the 3′-end of U6 snRNA by a series of open-to-closed conformation cycles of the catalytic domain (Munoz-Tello et al., 2014; Yates et al., 2015), the 3′-region of the oligo-uridylylated tail would be compressed within the active pocket of TUT1. Consequently, the 3′-end of U6 snRNA would no longer relocate to the active site. Finally, TUT1 terminates RNA synthesis and oligo-uridylylated U6 snRNA is released from the enzyme, as observed in the mechanism of RNA synthesis termination by tRNA nucleotidyltransferases (Tomita and Yamashita, 2014; Yamashita et al., 2014, 2015; Yamashita and Tomita, 2016).

### TUT1 Can Function as a PAP

While TUT1 has been originally identified as a U6 snRNA-specific TUTase (Trippe et al., 1998, 2003, 2006), TUT1 also reportedly functions as a PAP, acting on specific mRNAs under oxidative stress conditions (Mellman et al., 2008). TUT1 interacts with phosphatidylinositol 4-phosphate 5-kinase Iα (PIPKIα) and its PAP activity is also activated by phosphatidylinositol 4,5-bisphosphate (PIns4,5P2) *in vitro* (Mellman et al., 2008; Mohan et al., 2015). Upon oxidative stress, TUT1 is recruited into the CPSF complex for the polyadenylation of specific oxidative-stress response mRNAs (Mellman et al., 2008; Laishram and Anderson, 2010). The PAP activity of TUT1 is also activated by several protein kinases (Gonzales et al., 2008; Laishram et al., 2011; Mohan et al., 2015).

The structure of TUT1-ATP complex revealed that the adenine base forms only one hydrogen bond with His549 (Figure 3D). Biochemical analysis indicated that TUT1 has a lower affinity for ATP than for UTP *in vitro* (Yamashita et al., 2017). The interaction of TUT1 with other factors and/or its phosphorylation by several kinases might promote CPSF complex formation at specific mRNAs. Since the KA-1 domain of MARK-3 binds to phospholipids (Moravcevic et al., 2010), the mobile KA-1 domain of TUT1 might interact with PIns4,5P2. This interaction might regulate the activity or localization of TUT1, and TUT1 recruitment to the CPSF complex might induce allosteric structural changes of TUT1 nucleotide-binding pocket to accommodate ATP. Thus, TUT1 might be able to add poly(A) tails to specific mRNAs under specific biological conditions. Detailed mechanism of the alteration of the nucleotide specificity of TUT1 in specific biological processes awaits further study.

## TUT4 and TUT7: Uridylylation of *Pre–let-7*

### Biogenesis of *Let-7*

MiRNAs are small (21–25-nt) non-coding RNAs that function in gene silencing. Together with Argonaute proteins, miRNAs form RNA-induced silencing complex, and inhibit protein synthesis or induce mRNA degradation by base-pairing with target mRNAs (Braun et al., 2012). *Let-7* is a highly conserved miRNA, from nematode to human, and is known to regulate various cellular processes (Bussing et al., 2008; Thornton and Gregory, 2012). It regulates cellular proliferation by acting as a tumor suppressor. It also regulates cellular differentiation, development, and apoptosis, and is involved in glucose metabolism (Reinhart et al., 2000; Houbaviy et al., 2003; Takamizawa et al., 2004; Johnson et al., 2005; Tsang and Kwok, 2008; Zhu et al., 2011).

The synthesis of most miRNAs begins with the transcription of a primary miRNA transcript (pri-miRNA) by RNA polymerase II. Then, pri-miRNA is cleaved to become precursor miRNA (pre-miRNA) by Drosha. Pre-miRNA is exported to the cytoplasm by Exportin-5. In the cytoplasm, pre-miRNA is further processed by Dicer to produce mature miRNA, which functions in gene silencing (Ha and Kim, 2014).

**FIGURE 5:**
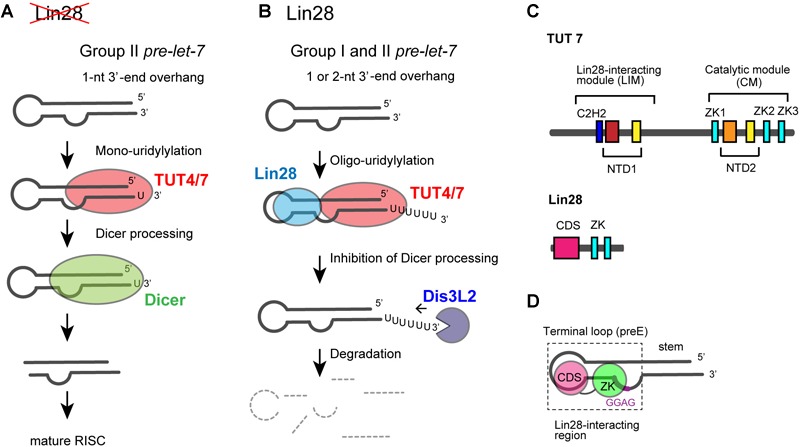
Functional duality of TUT4/7 in the biogenesis of *let-7*. **(A)** In the absence of LIN28, mono-uridylylation of *pre–let-7* that harbors 1-nt 3′-overhang (group II) by TUT4/7 promotes Dicer processing of *pre–let-7*. **(B)** In the presence of LIN28, TUT4/7 oligo-uridylylates *pre-let-7* and inhibits Dicer processing of *pre–let-7*. Oligo-uridylylated *pre–let-7* is degraded by DIS3L2, an exonuclease. **(C)** Schematic representation of domain organization of TUT4/7 and Lin28. ZKs in Lin28 interact with LIM of TUT4/7 in the presence of *pre–let-7* (Faehnle et al., 2017; Wang et al., 2017). **(D)** Schematic representation of the secondary structure of *pre–let-7* and interactions with Lin28B (Nam et al., 2011; Wang et al., 2017). ZK of Lin28 binds GGAG motif in *pre–let-7* and CDS binds the terminal loop of preE.

Among seven non-canonical terminal nucleotidyltransferase family proteins, TUT4 and TUT7 have similar domain organizations (Figure 1), and both are involved in the uridylylation of *pre–let-7*. Biogenesis of *let-7* is regulated by two distinct modes of uridylylation of *pre–let-7*: mono-uridylylation and oligo-uridylylation (Figures 5A,B).

Mono-uridylylation of *pre–let-7* is observed in differentiated and somatic cells where Lin28 is not expressed. Group II *pre–let-7* with 1-nt 3′-end overhang after Drosha processing is mono-uridylylated (Heo et al., 2012). This mono-uridylylation of *pre–let-7* is mediated by TUT4/7, and promotes subsequent Dicer processing, as *pre–let-7* with 2-nt 3′-overhang is a good substrate of Dicer. Thus, TUT4/7 promotes biogenesis of *let-7*, serving as a biogenesis factor (Figure 5A).

On the other hand, in embryonic cells and cancer cells, RNA-binding protein Lin28 is expressed and *let-7* expression is repressed. Lin28 binds to a conserved sequence (5′-GGAG-3′) in the loop region of *pre–let-7*after Drosha processing (Heo et al., 2008; Newman et al., 2008; Nam et al., 2011). Lin28 binding to *pre–let-7* competes with Dicer cleavage of *pre–let-7*, recruits TUT4/7, and promotes oligo-uridylylation of *pre–let-7* (Heo et al., 2008, 2009; Piskounova et al., 2008; Hagan et al., 2009; Thornton et al., 2012). Oligo-uridylylation of *pre–let-7* inhibits the Dicer processing and causes degradation of *pre-let-7* by DIS3L2 (Astuti et al., 2012; Ustianenko et al., 2013), an exonuclease that preferably degrades poly(U) tail. Hence, oligo-uridylylation of *pre–let-7* represses expression of mature *let-7* (Figure 5B). TUT4 is mainly responsible for the oligo-uridylytation of *pre-let-7*, because single knockdown of TUT4 is sufficient to increase the mature *let-7* levels (Heo et al., 2009; Thornton et al., 2012). On the other hand, TUT7 is thought to have limited or redundant role, because single knockdown of TUT7 has no effect on mature *let-7* level, but double knockdown of TUT4 and TUT7 increases mature let-7 more significantly than the single knockdown of TUT4 (Thornton et al., 2012). In the case of Lin28 dependent oligo-uridylylation, TUT4/7 serves as a negative regulator of *let-7* biogenesis, and contributes to tumorigenesis and embryonic stem cell maintenance, by canceling the repression of several genes.

The functional duality of uridylylation by TUT4/7 depends on the length of 3′-tail of *pre–let-7* and the cell type. Lin28 and TUT4/7 act as a molecular switch in the developmental and pathological transition observed in cancer.

### Domain Structures of Human TUT4/7 and Lin28

TUT4 and TUT7 have similar domain organization (Figure 1), and are multi-domain enzymes composed of an N-terminal ZF domain, two nucleotidyltransferase domains (NTD1 and NTD2) connected by a flexible linker, and three zinc knuckle domains (ZK) (CCHC-type zinc fingers in Figure 1). NTD1 in the N-terminal portion of the protein is not an active nucleotidyltransferase, since it lacks three catalytic carboxylates. By contrast, NTD2 has such three catalytic carboxylates and participates in the nucleotidyltransfer reaction (Figure 5C).

During the Lin28-dependent oligo-uridylylation of *pre–let-7* by TUT4/7, Lin28 and *pre–let-7* interact with the N-terminal half of TUT4/7 (Nam et al., 2011; Thornton et al., 2012; Faehnle et al., 2017; Wang et al., 2017). The N-terminal and C-terminal halves of TUT4/7 are referred to as LIM and CM, respectively (Figure 5C).

The molecular mechanism of Lin28 binding to *pre–let-7* RNA is well understood (Loughlin et al., 2011; Nam et al., 2011; Mayr et al., 2012; Wang et al., 2018). Human Lin28 harbors an N-terminal cold-shock domain, and two C-terminal ZKs. Cold-shock domain binds to a stem-loop structure in the pre-element (preE) and ZKs bind to a conserved GGAG motif located near the 3′-end of preE (Figure 5D). ZKs of Lin28 are necessary and sufficient for the ternary interactions of TUT4/7, Lin28, and *pre–let-7* (Faehnle et al., 2017; Wang et al., 2017).

### Structure of the Catalytic Core of TUT7 During Mono-Uridylylation

**FIGURE 6:**
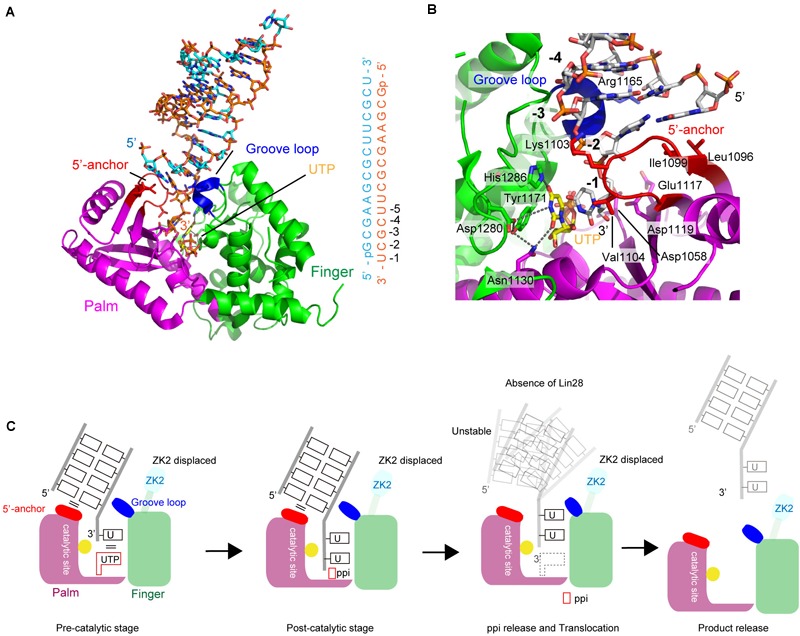
Structure of human TUT7 representing mono-uridylylation. **(A)** The overall structure of TUT7 CM complexed with dsRNA that mimics the double-helix of group II *pre–let-7* and UTP. ZK2 was not visible in the structure suggesting that the ZK2 is displaced. **(B)** UTP recognition by TUT7. UTP is depicted by sticks. **(C)** Schematic representation of mono-uridylylation of *pre–let-7* with 1-nt 3′-overhang (group II). After mono-uridylylation of group II *pre–let-7* and pyrophosphate (ppi) release, *pre–let-7* tanslocates. In the absence of Lin28, the mono-uridylylated *pre–let-7* cannot form a stable complex with TUT7, and is easily released from TUT7.

Recently, crystal structure of a complex of human TUT7 CM with 14-bp palindromic dsRNA and UTP was reported (Faehnle et al., 2017). The RNA used for crystallization contained a 1-nt 3′-overhang, thus mimicking the duplex stem of group II *pre–let-7* (Heo et al., 2012). Hence, the structure of CM in complex with dsRNA reflects mono-uridylylation of *pre–let-7* (Figure 6A).

The overall structure of TUT7 CM shares topological homology with those of yeast Cid1 and vertebrate mitochondrial PAP (Bai et al., 2011; Lunde et al., 2012; Munoz-Tello et al., 2012; Yates et al., 2012; Lapkouski and Hallberg, 2015). It is also homologous to the catalytic core structure of human TUT1 (Yamashita et al., 2017), and consists of palm and finger domains. TUT7 (and TUT4) CM contains ZKs (Figures 1, 5C). However, ZK2 is not visible in the structure, suggesting that it is displaced. In the structure of CM with dsRNA and UTP, UTP resides at the bottom of the cleft between palm and fingers, as observed in the structure of human TUT1 complexed with UTP. The O_4_ atom and O_2_ atom of UTP form hydrogen bonds with His1286 and Asn1130, respectively. The N_3_ atom of UTP interacts with Asp1280 via a water molecule (Figure 6B). The mechanism of UTP selection by human TUT7 (and TUT4) is essentially the same as that for yeast Cid1 and human TUT1 (Figure 3D).

The dsRNA, mimicking the duplex stem of *pre–let-7*, is clamped by two regions: the 5′-anchor and groove loop (Figures 6A,C). Leu1096 and Ile1099 in the 5′-anchor (palm) provide a hydrophobic platform for interactions and stack with the first-base pair of dsRNA. The groove loop (fingers) interacts with the minor groove of dsRNA through van der Waals interactions and hydrogen bonding. Consequently, the 3′-end overhanging nucleotide (3′-U) of group II pre-miRNA can enter the catalytic pocket. The uracil base of 3′-U is sandwitched between the uracil base of incoming UTP in the catalytic site and Val1104 in the 5′-anchor. The structure represents the pre-reaction stage of monouridylation. Following the nucleotidyltransfer reaction, the release of byproduct, pyrophosphate, would trigger the translocation of the double helix of *pre–let-7*, with the double helix of *pre–let-7* no longer fixed or stabilized by the 5′-anchor and groove loop. Consequently, TUT7/4 would terminate the mono-uridylylation reaction and release mono-uridylylated *pre–let-7* (Figure 6C).

### Structure of the Catalytic Core of TUT7 During Oligo-Uridylylation

**FIGURE 7:**
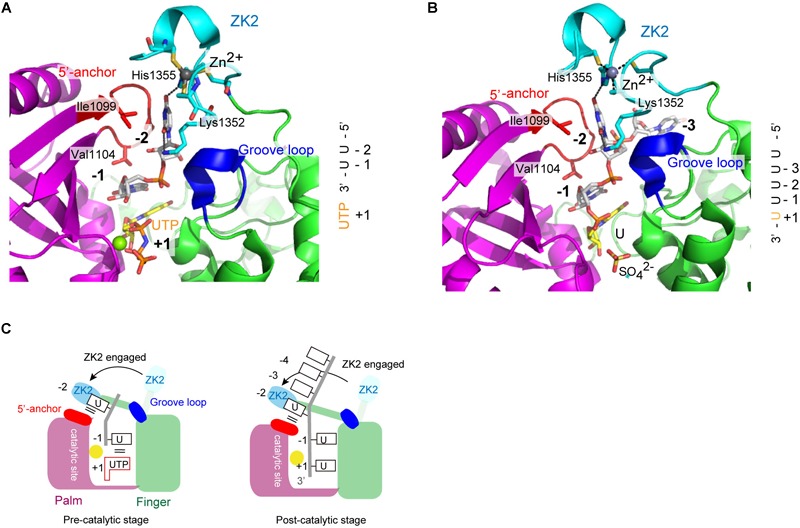
Structure of human TUT7 reflecting oligo-uridylylation. **(A)** Structure of TUT7 CM complexed with UUOH and UTP, representing the pre-catalytic stage. **(B)** Structure of TUT7 CM complexed with UUUUUOH (U5), representing the post-catalytic stage. **(C)** Schematic representation of oligo-uridylylation of *pre–let-7* with 2-nt 3′-overhang (group II). ZK2 participates in the recognition of uridine at position -2 to stabilize the 3′-oligo(U) in the pre- (left) and post- (right) catalytic stages.

Structure of human TUT7 CM complexed with 2-nt oligo(U) (5′-UUOH-3′) and a UTP analog, reflecting oligo-uridylylation (pre-catalytic stage), was reported (Faehnle et al., 2017; Figure 7A). Structure of a TUT7 CM in complex with a 5-nt oligo(U) (5′-UUUUUOH-3′), reflecting post-uridylylation (post-catalytic stage) was also reported (Figure 7B). In the structures of the CM-oligo(U) complex, ZK2 is clearly visible and interacts with the oligo(U) chain. In the structure of the CM-dsRNA complex representing mono-uridylylation, ZK2 is not visible and is displaced because of the presence of dsRNA (Figure 6A).

In both CM-oligo(U) complexes, ZK2 interacts with uridine at a position corresponding to -2 (Figures 7A,B). The O_4_ atom of uridine at position -2 forms a hydrogen bond with His1355, and the O_2_ atom of uridine at position -2 forms a hydrogen bond with a water molecule, which also hydrogen-bonds with Lys1353. The uridine at position -1 also hydrogen-bonds with Asn1124, and the uracil base is sandwitched between uracil base at position + 1 and Val1104. Thus, ZK2 would stabilize the oligo(U) reaction product and aid the translocation of oligo(U) via uracil-specific interactions at the oligo-uridylylation site (Figure 7C).

### Mechanism of Switching Between Mono- and Oligo-Uridylylation

**FIGURE 8:**
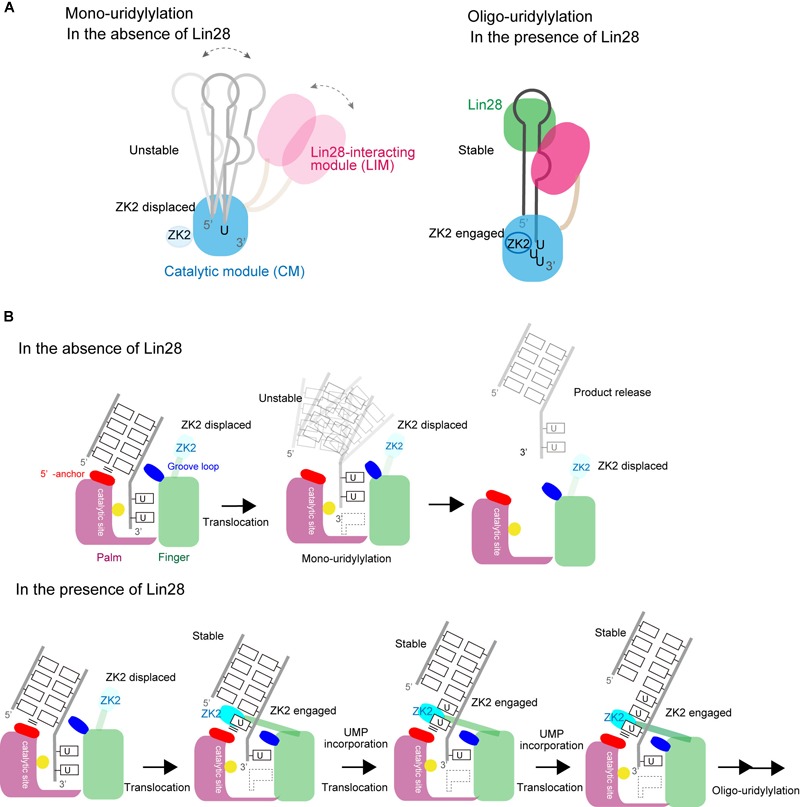
Switching between mono- and oligo-uridylylation. **(A)** Schematic representation of mono-uridylylation in the absence of Lin28 (left) and oligo-uridylylation in the presence of Lin28 (right). **(B)** Schematic detailed representations of mono-uridylylation of *pre–let-7* in the absence of Lin28 (upper), and oligo-uridylylation of *pre–let-7* in the presence of Lin28 (lower). Only the catalytic site in the CM is presented.

A TUT7/4 activity switch has been proposed based on the structures of TUT7/4 CM in complex with various RNAs (Figures 8A,B). Transient interaction between TUT7/4 and group II *pre–let-7* favors addition and release before oligo-uridylylation occurs. Hence TUT7/4 mono-uridylylate group II *pre–let-7* (Figure 8A). Since group I *pre–let-7* with a 2-nt 3′-overhang binds at the post-catalytic state, *pre–let-7* is released without oligo-uridylylation. The double-stranded stem of *pre–let-7* prevents ZK2 engagement in the process.

Lin28 controls the oligo-uridylylation switch by recruiting TUT7/4 LIM to the GGAG motif in the terminal loop of *pre–let-7* (Figure 8B). The stable ternary complex of TUT7/4, Lin28, and *pre–let-7* allows the 3′-end of *pre–let-7* to stay in the active site in CM, and supports processive oligo-uridylylation by the CM. During oligo-uridylylation, ZK2 in the CM interacts with 3′-oligo(U) tail and stabilizes the translocation of oligo(U) tail.

It is not yet clear how LIM interacts with ZK of Lin28 and the GGAG motif of *pre–let-7*, and how the interaction relocates the 3′-end of *pre–let-7* in the catalytic pocket of CM to initiate oligo-uridylylation. Elucidation of these mechanisms awaits further structural analysis.

## Perspectives

TUT1 participates in the target RNA-directed miRNA degradation, TRDM (Haas et al., 2016), where TUT1 oligo-uridylylates specific miRNAs for degradation by DIS3L2. TUT4 and TUT7 also oligo-uridylylate histone mRNAs for degradation after the inhibition of DNA replication (Schmidt et al., 2011; Lackey et al., 2016). They also oligo-uridylylate Ago cleaved pre-miRNAs with 5′ overhangs (Liu et al., 2014). Similarly, TUT4/7 oligo-uridylylates 3′-end of polyadenylylated mRNAs and marks them for degradation (Lim et al., 2014; Morgan et al., 2017; Chang et al., 2018). TUT4/7 uridylylates mature miRNAs (Jones et al., 2009; Jones et al., 2012; Thornton et al., 2015), which blocks miRNA activity, probably by affecting either the target specificity or RNA-induced silencing complex loading (Jones et al., 2012). Thus, RNA uridylylation by TUTases plays important roles in various aspects of gene expression. The molecular mechanisms of specific RNA substrates by TUTases remain elusive and cannot be fully explained by the currently solved structures. TUTases would recognize various RNAs either directly or through the regulatory factors which assist TUTases in recognizing specific RNA species. Elucidation of the regulatory mechanism of specific RNA uridylylation by TUTases awaits further study.

## Author Contributions

YY and KT wrote the manuscript together.

## Conflict of Interest Statement

The authors declare that the research was conducted in the absence of any commercial or financial relationships that could be construed as a potential conflict of interest.

## Abbreviations

CM: catalytic module
CPSF: cleavage and polyadenylation specificity factor
dsRNA: double-stranded RNA
ISL: internal stem-loop
KA-1: kinase associated-1
LIM: Lin28-interacting module
miRNA: microRNA
PAP: poly(A) polymerase
RRM: RNA recognition motif
snRNA: small nuclear RNA
snRNP: small nuclear ribonucleoprotein
TUTase: terminal uridylyltransferase
ZF: zinc finger
ZK: zinc knuckle
